# Cryopreservation of *Abies alba* × *A. numidica* and *Pinus nigra* embryogenic tissues by stepwise dehydration method

**DOI:** 10.1186/s13007-023-01131-w

**Published:** 2024-01-17

**Authors:** Teresa Hazubska-Przybył, Mikołaj Krzysztof Wawrzyniak, Agata Obarska, Terezia Salaj

**Affiliations:** 1 Department of Developmental Biology, Institute of Dendrology, Polish Academy of Sciences, Parkowa 5, 62-035 Kórnik, Poland; 2 Institute of Plant Genetics and Biotechnology Plant Science and Biodiversity Centre Slovak Academy of Sciences, Akademicka 2, P.O. Box 39A, Nitra, 950-07 Slovakia

**Keywords:** *Abies* hybrids, Pine, Somatic embryogenesis, Osmotic dehydration, Ex situ conservation

## Abstract

**Background:**

Cryopreservation makes it possible to preserve plant biodiversity for thousands of years in ex situ storage. The stepwise dehydration method is a simple and versatile cryopreservation technique based on the vitrification phenomenon. However, the commonly used dimethyl sulfoxide (DMSO) in this cryopreservation technique is considered harmful for plant material, thus alternative methods are needed to be applied.

**Results:**

In this study, the possibility of cryopreservation of embryogenic tissues (ETs) of *Abies alba* x A. *numidica* and *Pinus nigra* was investigated. Before freezing, ETs were partially dehydrated in the presence of increasing concentrations of sucrose (from 0.25 to 1.0 M) for 7 days, followed by desiccation of the tissues over silica gel for 2 and 2.5 h, respectively. After these pretreatments, the plant material was frozen in liquid nitrogen (LN; –196 °C). For both coniferous trees the ET survival rate was high and reached 84.4% for *A. alba* x *A. numidica* (28 days) and 86.7% for *P. nigra* (35 days) after recovery of the tissues from liquid nitrogen (LN). The regenerated tissue of *A. alba* x *A. numidica* was characterized by more intense growth after storage in LN compared to tissue that had not been cryopreserved (control). The tissue of this tree also undertook relatively rapid growth after thawing from LN. In turn, the ET growth of *P. nigra* was significantly lower after thawing compared to the other treatment.

**Conclusions:**

The present study demonstrated, that the stepwise dehydration method could be successfully applied to the cryostorage of ETs of both studied trees. To the best of our knowledge, this is the first report on ET cryopreservation based on this method for *Abies* and *Pinus* genus representatives, which may be the alternative way for efficient, long-term preservation of germplasm in LN.

## Background

Cryopreservation is a safe and cost-effective method for the long-term conservation of various plant materials, such as seeds, dormant buds, apices, embryogenic tissues (ETs), and somatic and zygotic embryos, of numerous plant species including trees [1–5]. It also does not induce genetic alterations and preserve the regeneration potential of the stored plant material [6]. Using this method, it is possible to preserve valuable gene resources of forest trees ex situ. It is especially important in the case of exceptional species, which are challenging or even impossible to conserve using conventional ex situ methods [7]. Thanks to cryopreservation, the morphogenetic potential and features of given cell lines are preserved, including the ability to produce specific secondary metabolites [8]. It is also possible to select genotypes resistant to biotic factors [9, 10]. Cryopreservation has also been used to eliminate viruses [11]. At the turn of the 1980s and 1990, in the experimental research into liquid nitrogen (LN) was introduced, a very diverse material from in vitro cultures, e.g. pollen [12], spores [13], shoot tips [14, 15], embryogenic cultures [16, 17]. In combination with somatic embryogenesis (SE), the method enables the production of somatic seedlings for commercial purposes and in pilot silvicultural programs, for example, in countries such as Canada, New Zealand, France, Sweden, and the United States [18–22]. SE is the most suitable micropropagation method used for commercial reforestation. It allows to obtain better results compared to organogenesis and rooted cuttings; therefore, it is applied to accelerate the tree breeding cycle and to produce clonal trees from improved individuals [23]. In conifers, ETs are the most suitable for cryopreservation because they are actively growing structures composed of cells that can be easily suspended in liquid [23]. Therefore, the most valuable genotypes of given species of tree can be selected and stored in gene banks in the form of ETs for further use in breeding programs. The cryostored tissues may be used at any time, and produce somatic seedling under in vitro culture conditions.

Embryogenic cultures of conifers are cryostored using (mainly) the slow-freezing method [24–28]. However, currently, other cryopreservation techniques are available, for example, encapsulation-dehydration and vitrification [29]. In vitrification-based procedures, plant material is exposed to concentrated cryoprotective media and/or air-dried and then frozen rapidly in LN. As a result, the water content of the cells is reduced to a level that allows the safe freezing of the material at ultralow temperatures, without the risk of ice crystal formation in the cells. Thus, the critical stage in these methods is the dehydration, not the freezing stage. The plant material should be amenable to decreasing the tissue's water content. Moreover, these methods are less complicated compared to classical methods. They do not require special equipment such as controlled freezer, and can be applied more widely, for different cell types. Pre-growth dehydration is one of the eight vitrification-based procedures described by Engelmann [29]. It has been successfully applied to oil palm polyembryonic cultures, coconut zygotic embryos, asparagus stem segments and pedunculate oak somatic embryos [30, 31]. ETs of *Picea abies* (L.) H. Karst and *P. omorika* (Pančić) Purk. have also been maintained in LN with good results using this simple cryopreservation method [3, 32]. The main advantage of this procedure is that it does not require a programmable freezer, expensive equipment, or the use of dimethyl sulfoxide (DMSO), which is considered harmful to plant material stored in LN. For example, Finkle et al. [33] claimed that DMSO used at a concentration of 2–10% may contribute to genetic, epigenetic, and other changes in cells of higher plants.

Silver fir (*A. alba* Mill) and Black pine (*P. nigra* Arn.) are the conifers trees that are native to Central and, in case of pine, also southeastern Europe. Due to the effect of higher pollution in the environment, the populations of silver fir have declined, for example in Slovakia. During the past decades, an extensive hybridization program was running with the aim of developing intra- and interspecific hybrids of *Abies* [34–37]. During the program, hybrid seeds were obtained, and except for the following development of hybrid seedlings in soil, attention has been paid to modern in vitro methods of plant propagation, predominantly somatic embryogenesis. Embryogenic tissues have been initiated in immature and mature zygotic embryos. The tissues have been successfully maintained under in vitro conditions, and after maturation of early somatic embryos, somatic seedlings were obtained [38–41].

*P. nigra* is a South European pine introduced to Slovakia in the past. The occurrence of individual trees or isolated stands may be found throughout the entire territory of the country. It has been reported the growth potential and output of the mentioned stands are comparable to those of several native species, such as *Fagus sylvatica*, *Carpinus betulus,* or *Quercus cerris* [42]. The *P. nigra* trees are recommended as pioneer trees convenient for recultivation of degraded or devastated soils and or bioindicators of environmental pollution [43]. Somatic embryogenesis and somatic seedlings regeneration in *P. nigra* was initiated repeatedly in several seasons [44–46].

The aim of our investigations was to verify whether the pre-growth dehydration method we developed for two *Picea* spp. [3, 32] will also be efficient in other coniferous trees such as *Abies* hybrid and *P. nigra* Arn. We tested the ETs of the *Abies alba* x *numidica* hybrid and *P. nigra.* In this study, we described the successful cryopreservation of both trees using the above mentioned method.

## Methods

### Plant material

Female strobili of silver fir (*A. alba* Mill.) growing in a natural forest stand in Slovakia were pollinated with pollen of Algerian fir (*A. numidica* de Lannoy ex Carrière) in early May. The cones containing hybrid seeds were collected during July and August, and the green cones of *P. nigra* in the first half of June. Geographical coordinates at the nature stand are: Latitude 48°18´ 35.61 “N, Longitude 18° 5´9.15 “E, and altitude 400 m. After collection, the cones were stored in paper bags in the refrigerator at 4 °C for 6–7 days. The seeds were excised from cones and surface-sterilized with 10% H_2_O_2_ for 10 min and rinsed 3–4 times in sterile distilled water. The integument was removed, and megagametophytes containing immature zygotic embryos were used as explants. For initiation of somatic embryogenesis, immature seeds isolated from green cones collected 2–3 months after pollination were used. The initiation of the AN72 cell line was done according to the protocol Salajova et al. [39]. Shortly, the isolated megagametophytes were placed on the surface of solid SH medium [47] containing 6-benzyladenine (BA) as the sole plant growth regulator. The initiated embryogenic tissue was maintained on the same solid medium for 6 years in darkness at 22 °C, following, the SH medium was replaced by medium DCR (containing the same plant growth regulator), elaborated specially for in vitro cultivation of conifer tissues [48]. The cell line AN72 was chosen for experiments due to the high proliferation rate on solid medium and maturation capacity. During the long-time cultivation, the production of cotyledonary somatic embryos was monitored [49]. The cell line produced well developed cotyledonary somatic embryos during the cultivation period.

In turn, the cell line E314 was initiated from an immature zygotic embryo enclosed in megagametophyte explant. According to microscopic observations, the zygotic embryo at the time of green cones collection and initiation was in the precotyledonary developmental stage [50]. The initiation medium DCR [47] was supplemented with 9 µM 2,4-D (2,4-dichlorophenoxyacetic acid) and 2.2 µM BA. The initiated tissue (E314) was white and mucilaginous consistency. Microscopic observations of the tissue revealed bipolar structures—somatic embryos typical for conifers. The cell line was vigorously growing and maintained on a solid DCR medium, the same as for initiation. Both induction and proliferation were performed in darkness at 22 °C.

### Cryopreservation protocol

ETs of *A. alba* x *A. numidica* and *P. nigra* were treated for 7 days with increasing concentrations of sucrose (0.25 M for 24 h; 0.5 M for 24 h; 0.75 M for 2 days, and 1 M for 3 days) in DCR solid medium. After this treatment, ETs were air-dried over silica gel (at 25 °C) for 2 and 2.5 h, respectively. Clumps of ET (the average weight of a clump with a diameter of 0.5 cm was about 0.200 g) were placed on the sterile nylon mesh cloth, sterilized in an autoclave (5 clumps per Petri dish with a 90-mm diameter) over silica gel (30 ml of gel per 90-mm Petri dish). The Petri dishes were closed, parafilm—wrapped, and placed in an incubator. The water content of plant material after cryoprotection alone and after both cryoprotection and air desiccation was determined before freezing ETs in LN according to [3]. As a control, ETs proliferated under in vitro culture conditions for 7 days without any treatments. After desiccation, ETs of *A. alba* x *A. numidica* and *P. nigra* were placed in empty, sterile 1.5-ml cryovials, rapidly frozen, and maintained in LN for 2 weeks or 24 h, respectively.

For cryopreservation of ET of *A. alba* x *A. numidica*, three Petri dishes with five pieces of tissue were used in each replicate. The experiment was repeated three times. A total of 240 clumps were tested for this *Abies* hybrid. For this hybrid, survival and regrowth were determined under control conditions (C) and after ET storage in LN (LN). In the case of *P. nigra* ET, four Petri dishes with five pieces were used. The experiment was conducted one time. A total of 75 clumps were tested. For black pine, the survival and regrowth were also measured during the preparation of tissue for freezing in LN, i.e., after ET treatment with sucrose (0.25–1.0 M; S) and after treatment with sucrose and then drying over silica gel (SSG).

### Recovery after cryopreservation

To recover the tissues after cryopreservation, the samples were quickly thawed in a water bath at 42 °C. Next, the samples were rehydrated on the same media, however, with decreasing concentrations of sucrose. In the case of *A. alba* x *A. numidica*, the samples were treated with a solid medium supplemented with 1.0–0.25 M concentration of sucrose for 1.5 h for each concentration, and in the case of *P. nigra* with 0.75–0.25 M for each dose of sucrose for 40 min. Then, ETs were placed on the proliferation medium for 4 weeks (*A. alba* x *A. numidica*) and for 5 weeks (*P. nigra*). During that time, the recovery rate and growth of ET in the one-week intervals were evaluated. During the last week, the level of tissue survival and mean regeneration time after recovery from LN were determined. The tissue survival was analyzed as binomial data, counting each growing clump as a success and the dead clump as a failure. Subsequently, logistic regression was estimated to compare each treatment. The weight of the ET samples growing in vitro after 0, 7, 14, 21, and 28 days of proliferation were measured to observe the latter growth. ET clumps with a diameter of approx. 0.5 cm and an average weight of approx. 0.200 g (both parameters were measured at the beginning of the experiments) after thawing were placed on the proliferation medium and weighed at specified time intervals using a laboratory scale from Sartorius AG Göttingen Germany type BP 301S. The means of three (*A. alba* x *A. numidica*) or four replicates (*P. nigra*) are reported values.

### Microscopic analysis

The fluorescein diacetate (FDA) test was used to determine the viability of recovered ET of *A. alba* x *A. numidica* and *P. nigra.* Surviving cells were visible as bright yellow–green colour under UV illumination (confocal microscope Leica SP 5). During proliferation, the ETs were stained with acetocarmine (2%) and observed under a light microscope (AxioVisionL rel. 4.8 program, New York, NY, USA, using a 5 × lens) to check the presence of early-stage somatic embryos in cryopreserved tissues.

### Statistical analysis

Data were analysed using R statistical computing software [51]. The regrowth of ET after freezing at LN was analysed using logistic regression binary distribution (growth—success, dead—failure). The data were checked for overdispersion using the DHARMa package [52]. ET growth was analysed using two-factor ANOVA (levels: time and treatment). Assumptions of ANOVA were checked using diagnostic plots. Post hoc analysis was performed using Tukey's test with the emmeans package [53].

All methods were performed in accordance with the relevant guidelines and regulations.

## Results

ETs of *A. alba* x *A. numidica* and *P. nigra* were successfully cryopreserved by a pre-growth dehydration method. In this method, the gradual desiccation of fir hybrid ET resulted in a decrease in water content from 96 to 70% after increasing the concentration of sucrose treatment, and it reached 31.6% before freezing ET in LN (after sucrose treatment and desiccation over silica gel; Table 1). In the case of *P. nigra,* the treatment of the tissue with increasing doses of sucrose-containing media resulted in 67.7% water content in ET, and the subsequent desiccation of the tissue over silica gel resulted in 27% water content (Table 1). We found a statistically significant relationship between the two desiccation treatments and the control in terms of changes in the tissue water content of both tested tree species.

**Table 1:** Water content of *A. alba* x *A. numidica* and* P. nigra* ETs after treatment with the cryoprotective media and after 2 or 2.5 h of air desiccation over silica gel

	Preculture	ET moisture content, %
*A. alba* × *A. numidica*	Control	96.0 ± 0.397 a
	Sucrose	70.1 ± 0.273 b
	Sucrose + Silica Gel	31.6 ± 0.042 c
*Pinus nigra*	Control	96.1 ± 0.088 a
	Sucrose	67.7 ± 0.408 b
	Sucrose + Silica Gel	27.0 ± 0.755 c

Mean ± standard error; p ≤ 0.05; Tukey’s test

**Fig. 1:**
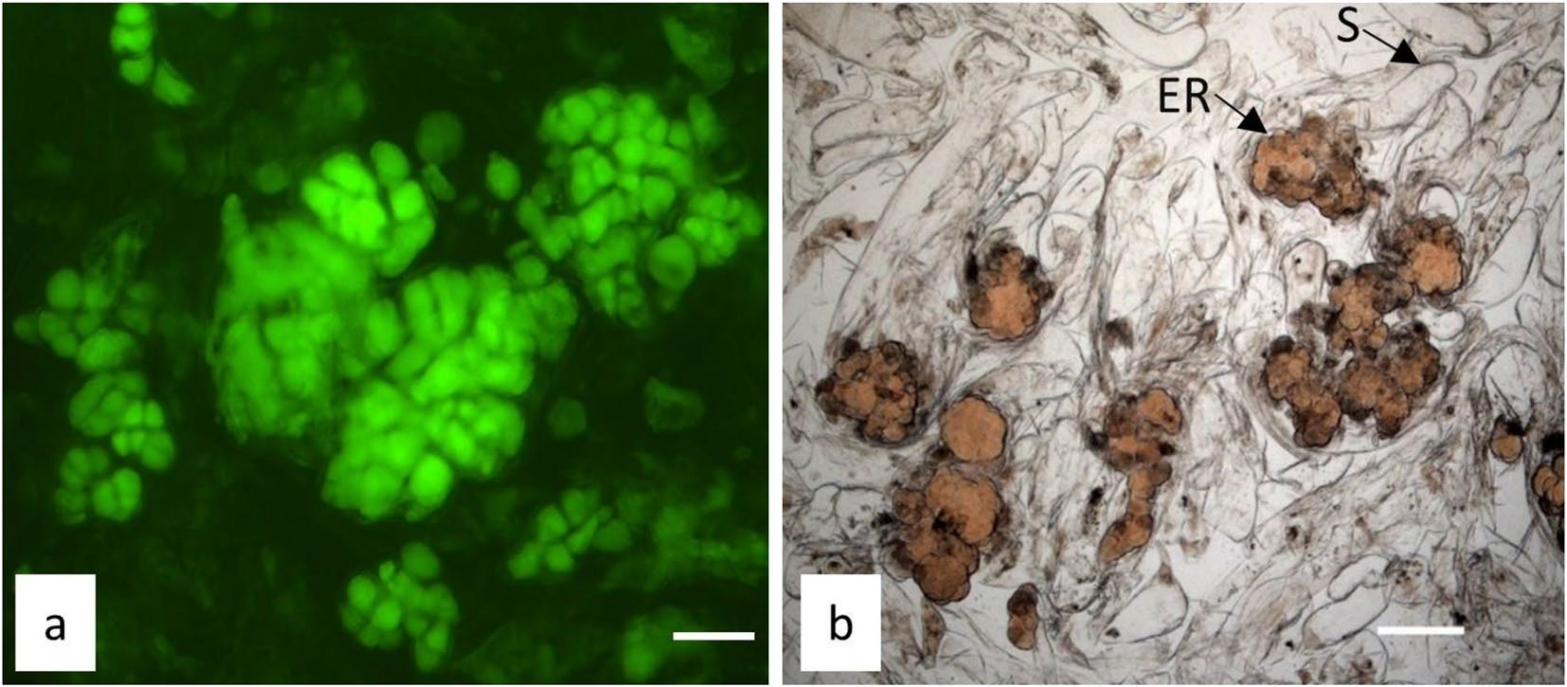
Viable cells of embryogenic regions of early-stage somatic embryos of *P. nigra* visible after staining the cryostored tissues based on the fluorescein diacetate (FDA) test (**a**). Early-stage somatic embryos of *P. nigra* (**b**) observed after acetocarmine staining (ERembryogenic region; Ssuspensor). Bars = 200 µm

Directly after thawing of *A. alba* x *A. numidica* and *P. nigra* ET, the FDA test demonstrated that cells from the embryonic regions of early-stage somatic embryos in cryopreserved ET survived freezing in LN (Fig. 1a). In contrast, the suspensors were not visible because they were destroyed during freezing in LN. The later staining of ETs with acetocarmine, after proliferation for a few weeks on DCR medium, revealed the presence of fully formed early-stage somatic embryos with embryogenic regions and suspensors (Fig. 1b), which regenerated after cryopreservation by the tested method.

**Fig. 2:**
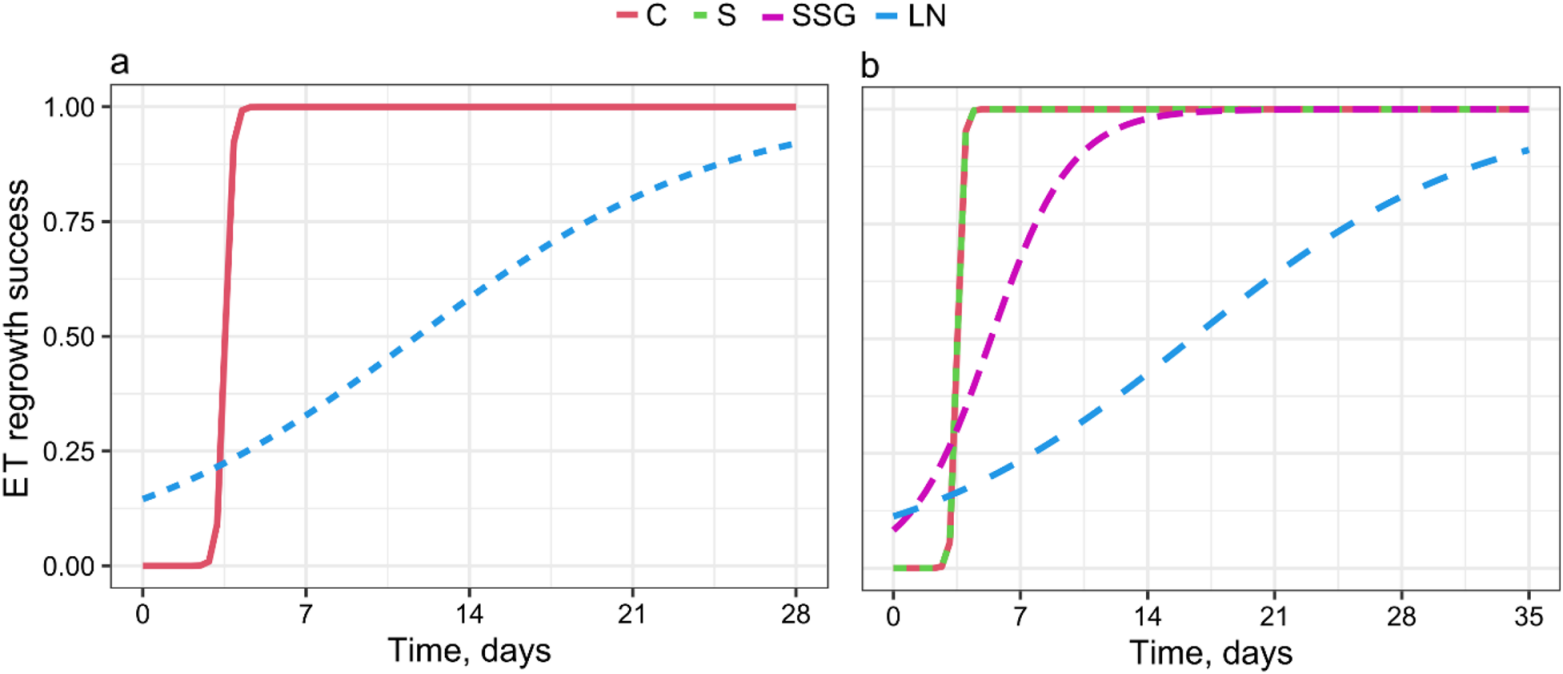
Logistic model of regrowth success of *A. alba* x *A. numidica* (**a**) and *P. nigra* (**b**) ETs during particular cryopreservation steps, C—Control—fresh ET, not cryopreserved; S—ET after treatment with sucrose (0.25–1.0 M) for 7 days; SSG—ET after treatment with sucrose (0.25–1.0 M) for 7 days and after air desiccation over silica gel (2 h in *A. alba* x *A. numidica* and 2.5 h in *P. nigra*); LN—ET after stepwise desiccation, freezing and thawing from LN

**Fig. 3:**
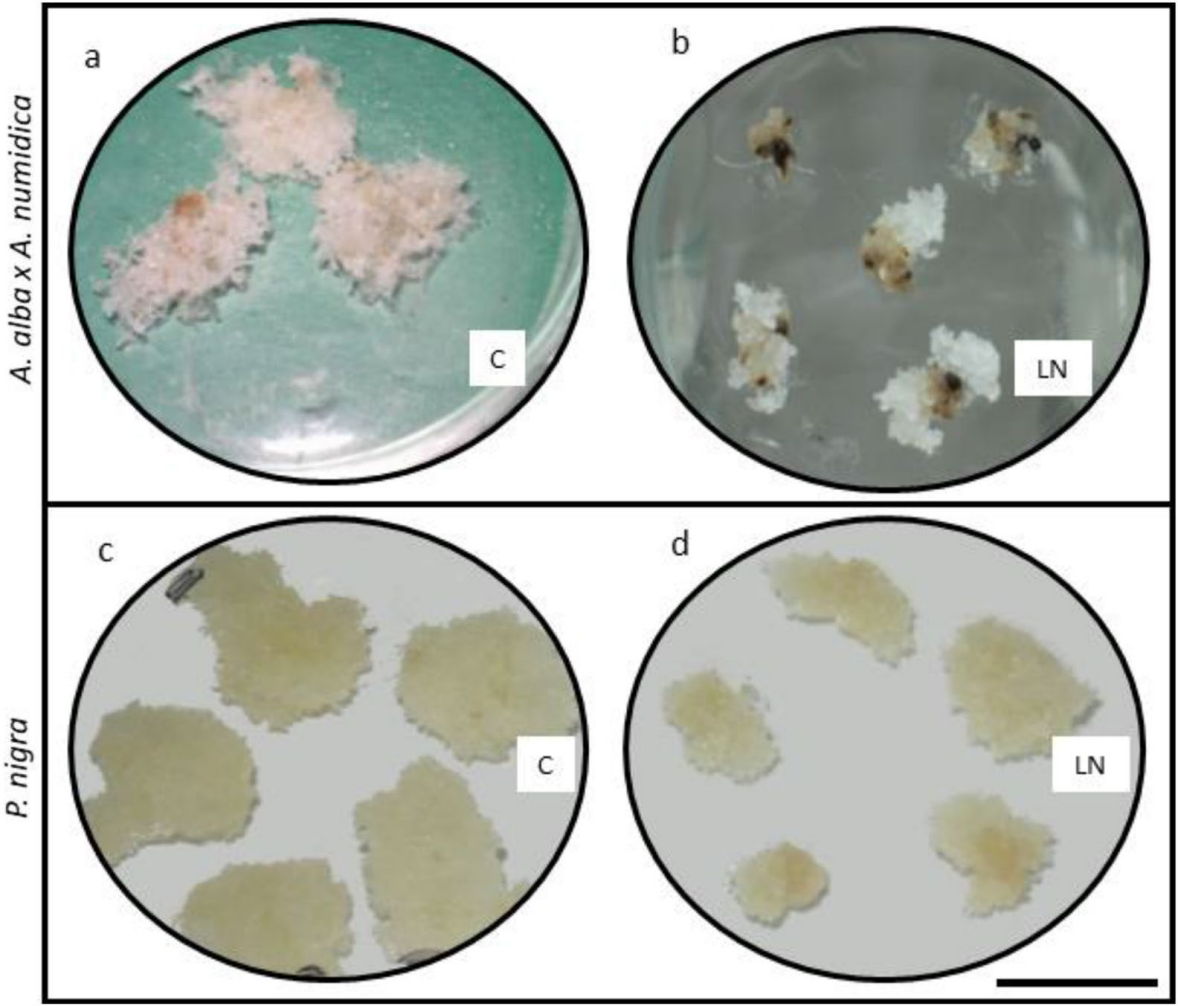
Regrowth of thawed ET clumps of *A. alba* x *A. numidica* and *P. nigra* in 12 and 28 days of in vitro culture, respectively. C—Control = fresh, non-cryopreserved ET (**a** and **c**); LN—ET after storage in LN (**b** and **d**). Bar = 10 mm

The thawed tissue of *A. alba* x A. *numidica* was characterized by more intense growth compared to tissue that had not been cryopreserved. The tissue of this tree undertook relatively rapid growth after thawing from LN. The mean regrowth time was 12 days after thawing (Fig. 2a and Table 2), while the ET of *P. nigra* was 17.9 days (Fig. 2b and Table 2). At that time, we observed the first characteristic white ‘flocs’ of tissue on the surface of the smooth clumps (Fig. 3) in both tree species. It was noticed that ETs from the control treatments regenerated during 7 days on average (Fig. 2a and Table 2). In the case of *P. nigra,* after sucrose pretreatment and air desiccation, regeneration took 7–9 days (Fig. 2b and Table 2). After cryopreservation, the ET regrowth rate was slower, however during subsequent weeks gradually increased in both tested coniferous trees. Finally, the survival reached 84.4% for *A. alba* x *A. numidica* and 86.7% for *P. nigra* after 28 and 35 days of recovery of ETs from LN, respectively (Table 2).

**Table 2:** *A. alba* x* A. numidica* and *P. nigra* ET survival after 28 and 35 days of growth on proliferation medium, respectively

Species	Preculture	ET regrowth, %	Mean regrowth Time, days
*A. alba x A. numidica*	C	100.0 ± 0.00 a	7.0 ± 0.00 a
	LN	84.4 ± 2.94 a	12.0 ± 0.63 b
*P. nigra*	C	100.0 ± 0.00 a	7.0 ± 0.00 a
	S	100.0 ± 0.00 a	7.0 ± 0.00 a
	SSG	100.0 ± 0.00 a	8.8 ± 1.75 a
	LN	86.7 ± 13.33 a	17.9 ± 4.21 b

C—Control—fresh ET, not cryopreserved; S—ET after treatment with sucrose (0.25–1.0 M) for 7 days; SSG—ET after treatment with sucrose (0.25–1.0 M) for 7 days and after 2 (*A. alba* x *A. numidica*) or 2.5 h *(P. nigra)* air desiccation over silica gel; LN—ET after stepwise desiccation, freezing and thawing from LN. Mean ± standard error; p ≤ 0.05; Tukey’s test

**Fig. 4:**
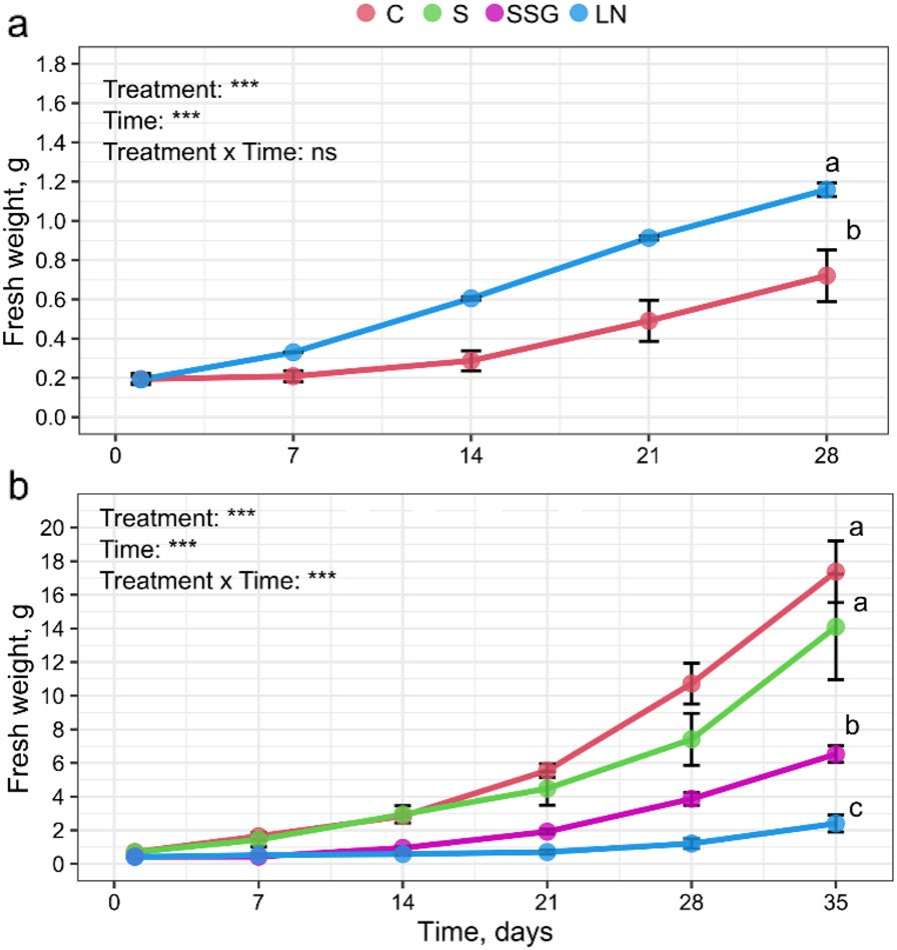
Growth of *A. alba* x *A. numidica * (**a**) and *P. nigra* (**b**) ET lines during 28 days and 35 days, after recovery from LN, respectively. C—Control = fresh, non-cryopreserved ET; S—ET treated with sucrose and SSG—ET treated with sucrose and silica gel (only *P. nigra* measured); LN—ET after storage in LN. Mean ± SE; ANOVA with Tukey’s test; ns p > 0.05; *: p ≤ 0.05; **: p ≤ 0.01; ***: p ≤ 0.001

The increase in *A. alba* x *A. numidica* tissue mass significantly depended on the treatment and time. While the interaction between these factors did not have a significant effect, and the weight increased at a similar rate in both tested treatments (Fig. 4a). However, surprisingly, ET growth was higher in the tissue stored in LN than in noncryostored tissue. In contrast, we observed an inverse relationship in the case of cryopreserved *P. nigra* ET (Fig. 4b). In this case, the ET mass growth was significantly higher for tissue from the control treatment (not subjected to osmotic and air dehydration) and for tissue pretreated with sucrose for 7 days than for tissue cryoprotected and stored in LN (Fig. 4b). The ability to grow ETs of both tree species after storage with the tested method indicates that the pre-growth dehydration method could also be used in the cryopreservation of genetic resources of conifers other than spruce species.

## Discussion

The obtained research results demonstrate that the pre-growth dehydration method is effective not only for spruce species [3, 32] but also for fir hybrid and black pine ET cultures. The survival rate for ET of *A. alba* x *A. numidica* (84.4%) and for *P. nigra* (86.7%) using this cryopreservation method was even higher than that obtained for ET of *P. abies*—54.4% [3]. Slightly better results of cryopreservation of ETs of *A. alba* x *A. numidica* (also cell line AN72) and *P. nigra* were obtained by Salaj et al. [24, 54], who used a slow freezing method. Those authors reported a 100% survival rate for all frozen lines of *A. alba* x *A. numidica* and 87.5% for seven out of eight frozen cell lines of *P. nigra*.

The slow freezing method is operationally complex because it requires the application of sophisticated and expensive programmable freezers [2]. In turn, pre-growth dehydration involves the desiccation of plant material before its freezing in LN using glucose or sucrose. According to Suzuki and coworkers [55], both sugars induce desiccation tolerance by osmotic dehydration. For example, induction of desiccation tolerance was reported for somatic embryos of *Medicago sativa* [56] or ET of *Quercus robur* [31] after pretreatment with a high sucrose concentration. This type of tolerance was also induced in ETs of *P. abies* and *P. omorika* in our previous investigations using a multistep approach [3, 32]. During osmotic dehydration, a simultaneous flux of water and solutes from and into cells occurs. Using this method, it is possible to reduce the water content in the plant material from 30 to 70% [57]. According to Yadav and Singh [57], osmotic dehydration is one the most suitable methods to increase the shelf life of fruits and vegetables and is often used before air desiccation.

ETs of *A. alba* x *A. numidica* and *P. nigra*, similar to both spruce species studied earlier, were characterized by high water content (96%), and they required its elimination to some extent to avoid its crystallization into ice before freezing in LN. Using multistep treatment and air-desiccation (2.5 or 2.0 h) gel, we reduced the water content of the tissues to 70.1 and 67.7% and then to 27 and 31.6% for *P. nigra* and *A. alba* x *A. numidica*, respectively. However, the final water content in these tissues was slightly higher than that in tissues of both spruce species [3, 32]. In these cases, the desiccation of the tissues for 2 h (after previous osmotic dehydration) resulted in a reduction of the water content to approximately 20%. The obtained results indicate that the ETs of *Abies* and *Pinus* differ slightly from those of the tested spruces because they are slightly less susceptible to water loss. This difference is probably due to differences in the occurrence of the same metabolites, e.g., sugars, in the cells or due to the differences in the structure of the cell walls of ET cells in these trees. Despite this, we eventually obtained the successful cryopreservation of tissues dehydrated to this rate. In order to simplify the cryopreservation procedure applied for *Picea* spp. [3, 32], we introduced two modifications to the previously used procedure at the stage of hydration of *P. nigra* tissue after its thawing from cryostorage. Namely, the thawed ET was transferred directly to the medium with 0.75 M sucrose concentration, omitting the 1.0 M concentration. In addition, the dehydration time of ET on individual concentrations was reduced to 40 min instead of 1.5 h, as was routinely done. The methodological modifications applied during ET rehydration did not reduce either ET survival or its regrowth under in vitro culture. We, therefore suppose that for some ET lines of coniferous species, it will be possible to speed up the cryopreservation process at this procedure stage.

ETs of both tree species started regrowth after thawing at different times, although in the Control treatment, the mean regrowth time was the same (see Table 2). Even sucrose treatment and further air desiccation did not limit the *P. nigra* ET time regeneration. ET of *A. alba* x *A. numidica,* started the regrowth during 12 days, and ET of *P. nigra* during approx. 18 days of culture on the proliferation medium after thawing. In both cases, ETs were white and mucilaginous with characteristic ‘flocs’ on the surface of clumps, and they looked like before cryostorage (see Fig. 3). Recovered ETs were able to proliferate similarly to ETs of *Picea omorika* and *P. abies* in our previous studies [3, 32], although at the beginning, their growth was slow (see Fig. 2). At the 7 day of the maintenance on the proliferation medium, ET regrowth success was approx. 0.30 for *Abies* hybrid and approx. 0.25 for *P. nigra*. Much more intensive ET regrowth started after 21 and 28 days, respectively. For comparison, Salaj and coworkers [24, 54] reported that after the application of the slow-freezing method, the regrowth of ETs in both *A. alba* x *A. numidica* and *P. nigra* started approximately one week after thawing from LN and massive growth occurred 14 days after thawing. We suppose that the particular cell line E413 used in our present experiments requires a longer time for recovery.

Cryopreservation via the slow freezing method is the most commonly used method in conifers [6, 27, 58, 59]. However, in this method, dimethyl sulfoxide (DMSO) is used, which can cause some genetic alterations [33, 60, 61]. Furthermore, it is also believed that different genotypes may be susceptible to genetic variation to different degrees [62]. Therefore, the availability of other cryopreservation methods that are not based on the use of DMSO is, in our opinion, desirable, which is also partly justified by the results of the study obtained by Nawrot-Chorabik and Sitko for *A. nordmanniana* [63], confirming the possibility of cryopreservation of embryogenic cultures without the need to use this cryoprotectant. These authors showed that of the two cryoprotectants tested, abscisic acid (ABA) was more effective compared to DMSO. This is also consistent with the result we obtained for *P. abies*, where the use of ABA had a beneficial effect on the cryopreservation and regeneration of somatic embryos from cryopreserved tissue [3]. In the case of *A. nordmanniana*, temperature balancing during rapid thawing and freezing of samples also had a positive effect, which resulted in a significant increase in tissue survival rate compared to the slow freezing and thawing method. Salaj et al. [24, 54] demonstrated that although the slow freezing method was used for *P. nigra* and *A. alba* x A. *numidica* ET cryopreservation, an RAPD analysis did not detect any genetic changes; thus, these findings confirm the genetic fidelity of the studied ETs stored in this way. The pre-growth dehydration method we tested eliminates the need to use DMSO. Our previous research demonstrated that the method we tested did not result in any genetic modification in cryopreserved *P. abies* ETs or in somatic embryos derived from these tissues after analysing 5 microsatellite loci in DNA [3]. Thus, it may be used for cryopreservation of conifer ETs as an alternative to the slow freezing method if DMSO may have a detrimental effect on the cryopreserved material. However, at the present research stage, there is a need to verify whether DMSO truly causes changes in the genetic material of cryopreserved tissue based on the latest molecular techniques.

Long-term storage of embryogenic callus of coniferous trees using safe cryopreservation methods offers great opportunities both at the economic (testing of elite genotypes for the needs of clonal forestry, e.g., in terms of wood productivity, resistance to pests, etc.) and ecological level (preservation of biological diversity, based on unique genotypes). Selection can be carried out for the most valuable genotypes, including those with interesting breeding characteristics. It may also concern unfavourable biotic [9] and abiotic [64, 65] factors affecting trees. In previous experiments altogether, 25 cell lines of *P. nigra* were selected with the aim to test their regeneration ability after cryopreservation [66, 67]. Subsequently, the regenerated tissues were used in maturation experiments using abscisic acid (94 µM) and maltose 6 to 9% [45]. Out of tested cell lines, 11 of them produced mature somatic embryos, capable of germination with frequencies 10.8–61,6–70.5%. Similarly, for *Abies* hybrids, cell lines characterized with regeneration capacity to develop mature embryos and regenerate plantlets/somatic seedlings were selected [54]. The cryopreserved tissues showed high regeneration capacity and produced mature somatic embryos capable of further development [54].

In conclusion, the pre-growth dehydration method tested in this study appeared to be efficient for the cryostorage of ETs of coniferous species other than spruces. Thus, in our opinion, it may be used as an alternative to slow freezing, especially for ET genotypes, which would be susceptible to genetic changes, using this method.

## Data Availability

The datasets generated during and/or analyzed during the current study are available from the corresponding author on reasonable request.

## Abbreviations

BA: 6-Benzyladenine
DMSO: Dimethyl sulfoxide
2,4-D: 2,4-Dichlorophenoxyacetic acid
ET: Embryogenic tissue
LN: Liquid nitrogen
SE: Somatic embryogenesis

## References

[1] Häggman HM, Ryynänen LA, Aronen TS, Krajnakova J (1998). Cryopreservation of embryogenic cultures of Scots pine. Plant Cell Tiss Organ Cult.

[2] Engelmann F, Thorpe TA, Yeung EC (2011). Cryopreservation of embryos: an overview. Plant Embryo Culture Methods and Protocols.

[3] Hazubska-Przybył T, Chmielarz P, Michalak M, Dering M, Bojarczuk K (2013). Survival and genetic stability of *Picea abies* embryogenic cultures after cryopreservation using a pregrowth-dehydration method. Plant Cell Tiss Organ Cult.

[4] Ahn CH, Tull AR, Montello PM, Merkle SA (2017). A clonal propagation system for Atlantic white cedar (*Chamaecyparis thyoides*) via somatic embryogenesis without the use of plant growth regulators. Plant Cell Tiss Organ Cult.

[5] Varis S, Ahola S, Jaakola L, Aronen T (2017). Reliable and practical methods for cryopreservation of embryogenic cultures and cold storage of somatic embryos of Norway spruce. Cryobiology.

[6] Ozudogru EA, Lambardi M (2016). Cryotechniques for the long-term conservation of embryogenic cultures from woody plants. Methods Mol Biol.

[7] Pence VC, Meyer A, Linsky J, Gratzfeld J, Pritchard HW (2022). Defining exceptional species—a conceptual framework to expand and advance ex situ conservation of plant diversity beyond conventional seed banking. Biol Cons..

[8] Arora K, Rai M, Sharma A (2022). Tissue culture mediated biotechnological interventions in medicinal trees: recent progress. Plant Cell Tiss Organ Cult.

[9] Wang B, Wang RR, Cui ZH, Bi WL, Li JW, Li BQ (2014). Potential applications of cryogenic technologies to plant genetic improvement and pathogen eradication. Biotechnol Adv.

[10] Nawrot-Chorabik K, Marcol-Rumak N, Latowski D (2021). Investigation of the biocontrol potential of two ash endophytes against *Hymenoscyphus fraxineus* using in vitro plant–fungus dual cultures. Forests.

[11] Wang MR, Chen L, Teixeira da Silva JA, Volk GM, Wang QC (2018). Cryobiotechnology of apple (Malus spp.): development, progress and future prospects. Plant Cell Rep.

[12] Impe D, Ballesteros D, Nagel M (2022). Impact of drying and cooling rate on the survival of the desiccation-sensitive wheat pollen. Plant Cell Rep.

[13] Ballesteros D, Estrelles E, Ibars AM (2006). Responses of Pteridophyte spores to ultrafreezing temperatures for long-term conservation in germplasm banks. Fern Gazette.

[14] Fukai S, Goi M, Tanaka M (1991). Cryopreservation of shoot tips of *Caryophyllaceae* ornamentals. Euphytica.

[15] Tannoury M, Vintejoux C, Dereuddre J, Tannoury M, Vintejoux C, Dereuddre J (1995). Cryopreservation of carnation shoot-tips (*Dianthus caryophyllus* L.) by encapsulation-dehydration. Acta Bot Gallica..

[16] Barra-Jiménez A, Aronen TS, Alegre J, Toribio M (2015). Cryopreservation of embryogenic tissues from mature holm oak trees. Cryobiology.

[17] Gladfelter HJ, Johnston J, Wilde HD, Merkle SA (2021). Somatic embryogenesis and cryopreservation of *Stewartia* species. Plant Cell Tiss Organ Cult.

[18] Högberg KA, Ekberg I, Norell L, von Arnold S (1998). Integration of somatic embryogenesis in a tree breeding programme: a case study with *Picea abies*. Can J For Res.

[19] Grossnickle SC, Sutton BCS (1999). Applications of biotechnology for forest regeneration. New For.

[20] Park YS (2002). Implementation of conifer somatic embryogenesis in clonal forestry: technicalrequirements and deployment considerations. Ann For Sci.

[21] Lelu-Walter MA, Thompson D, Harvengt L, Sanchez L, Toribio M, Pâques LE (2013). Somatic embryogenesis in forestry with a focus on Europe: state-of-the-art, benefits, challenges and future direction. Tree Genet Genomes.

[22] Egertsdotter U, Ahmad I, Clapham D (2019). Automation and scale up of somatic embryogenesis for commercial plant production, with emphasis on conifers. Front Plant Sci.

[23] Charest P, Klimaszewska K, Bajaj YPS (1995). Cryopreservation of germplasm of *Larix* and *Picea* species. Cryopreservation of Plant Germplasm I.

[24] Salaj T, Matušíková I, Fráterová L, Piršelová B, Salaj J (2011). Regrowth of embryogenic tissues of *Pinus nigra* following cryopreservation. Plant Cell Tiss Organ Cult.

[25] Krajňáková J, Gömöry D, Häggman H, Ahuja MR, Ramawat KG (2014). Biotechnology tools for conservation of the biodiversity of European and Mediterranean *Abies* species. Biotechnology and Biodiversity, Sustainable Development and Biodiversity.

[26] Trontin JF, Teyssier C, Morel A, Harvengt L, Lelu-Walter MA, Bonga JM, Moon HK (2016). Prospects for new variety deployment through somatic embryogenesis in maritime pine. Y-S Park Vegetative Propagation of Forest Trees.

[27] Nunes S, Marum L, Farinha N, Pereira VT, Almeida T, Dias MC (2017). Plant regeneration from ploidy-stable cryopreserved embryogenic lines of the hybrid
* Pinus elliottii
* x
* P
*
. caribaea. Ind Crops Prod..

[28] Chen X, Jiang S, Dai J, Yuan D, Kong L, Zhang J (2021). Cryopreservation of embryogenic callus for *Larix gmelinii* var. *principis-rupprechtii*. J Beijing For University..

[29] Engelmann F (2004). Plant cryopreservation: Progress and prospects. In Vitro Cell Dev Biol Plant.

[30] Engelmann F, Engles J, Dullo E. The development of complementary strategies for the conservation of plant genetic resources using in vitro and cryopreservation methods. In: In Vitro Conservation and Cryopreservation of Tropical Fruit Species. New Delhi: IPGRI Office for South Asia and NBPGR; 2003. p. 37–48.

[31] Chmielarz P, March G, Boucaud MT (2005). Cryopreservation of *Quercus robur* L. embryogenic calli. Cryo-Lett.

[32] Hazubska-Przybył T, Chmielarz P, Michalak M, Bojarczuk K (2010). Cryopreservation of embryogenic tissues of *Picea omorika* (Serbian spruce). Plant Cell Tiss Organ Cult.

[33] Finkle BJ, Zavala ME, Ulrich IM. Cryoprotective compounds in the viable freezing of plant tissues. Florida: K.K. Kartha editor, CRC Press. Boca Raton; 1985.p. 75–113

[34] Kormut’ák A (1985). Study on species hybridization within the Genus *Abies*.

[35] Kormut’ák A, Vooková B, Gajdošová A, Salaj J (1992). Hybridological relationship between *Pinus nigra* Arn., *Pinus thunbergii* Parl. and *Pinus tabulaeformis* carrier. Silvae Genet..

[36] Kormuťák A, Vooková B, Salaj T, Čamek V, Galgóci M, Maňka P (2012). Crossability relationships between Noble, Manchurian and Caucasian firs. Acta Biol Crac Ser Bot.

[37] Kormuťák A, Vooková B, Čamek V, Salaj T, Galgóci M, Maňka P (2013). Artificial hybridization of some *Abies* species. Plant Syst Evol.

[38] Gajdošová A, Vooková B, Kormuťák A, Libiaková G, Dolezel J (1995). Induction, protein-composition and DNA-ploidy level of embryogenic calli of Silver fir and its hybrids. Biol Plant.

[39] Salajova T, Jasik J, Kormuťák A, Salaj J, Hakman I (1996). Embryogenic culture initiation and somatic embryo development in hybrid firs (*Abies alba* x *Abies cephalonica*, and *Abies alba* x *Abies numidica*). Plant Cell Rep.

[40] Vooková B, Gajdošová A, Matúšová R (1997). Somatic embryogenesis in *Abies alba* × *Abies alba* and *Abies alba* × *Abies nordmanniana* hybrids. Biol Plant.

[41] Salaj T, Salaj J (2003). Somatic embryo formation on mature *Abies alba* x *Abies cephalonica* zygotic embryo explants. Biol Plant.

[42] Tokár F (1985). The distribution of exotic woody plants in the forest stands of the Low Carpathians mountain and the ecological and production analyses of the main species. Lesnictví.

[43] Mičieta K, Murín G (1998). Three species of genus pinus suitable as bioindicators of polluted environment. Water Air Soil Poll.

[44] Salajova T, Salaj J, Jasik J, Kormut’ák A, Gupta PK, Newton RJ (1995). Somatic Embryogenesis in *Pinus nigra* Arn. Jain SM.

[45] Salajova T, Salaj J, Kormut’ák A (1999). Initiation of embryogenic tissues and plantlet regeneration from somatic embryos of *Pinus nigra* Arn. Plant Sci..

[46] Salajova T, Salaj J (2005). Somatic embryogenesis in *Pinus nigra*: Embryogenic tissue initiation, maturation and regeneration ability of established cell lines. Biol Plant.

[47] Schenk RU, Hildebrandt AC (1972). Medium and techniques for induction and growth of monocotyledonous and dicotyledonous plant cell cultures. Can J Bot.

[48] Gupta PK, Durzan DJ (1985). Shoot multiplication from mature trees of Douglas-fir (*Pseudotsuga menziesii*) and sugar pine (*Pinus lambertiana*). Plant Cell Rep.

[49] Salaj T, Matúšová R, Salaj J (2004). The effect of carbohydrates and polyethylene glycol on somatic embryo maturation in hybrid fir *Abies alba* × *Abies numidica*. Acta Biol Cracov Bot.

[50] Salaj T, Klubicová K, Matúšová R, Salaj J (2019). Somatic embryogenesis in selected conifer trees Pinus nigra Arn. and Abies hybrids. Front Plant Sci.

[51] R Core Team. R: A language and environment for statistical computing. Vienna, Austria. R Foundation for Statistical Computing; 2020.

[52] Hartig F. DHARMa: Residual Diagnostics for Hierarchical (Multi-Level / Mixed) Regression Models. R package version 0.4.5.2022.

[53] Lenth RV. Packed „emmeans”: Estimated Marginal Means, aka Least-Squares Means. R package version 1.7.2. 2022.

[54] Salaj T, Matušíková I, Panis B, Swennen R, Salaj J (2010). Recovery and characterisation of hybrid firs (*Abies alba x A. cephalonica, Abies alba x A. numidica*) embryogenic tissues after cryopreservation. Cryo-Lett..

[55] Suzuki M, Ishikawa M, Akihama T (1998). A novel preculture metod for the induction of desiccation tolerance in gentian axillary buds for cryopreservation. Plant Sci.

[56] Senaratna T, MacKersie B, Bowley ST (1989). Desiccation tolerance of alfalfa (*Medicago sativa* L.) somatic embryos. Influence of abscisic acid, stress pretreatments and drying rates. Plant Sci..

[57] Yadav AK, Singh SV (2014). Osmotic dehydration of fruits and vegetables: a review. J Food Sci Technol.

[58] Lambardi M, Ozudogru EA, Barberini S, Danti R (2018). Strategies for fast multiplication and conservation of forest trees by somatic embryogenesis and cryopreservation: a case study with cypress (*Cupressus sempervirens* L.). Not Bot Hort Agrobot..

[﻿59] Salaj T, Klubicová K, Matúšová R, Salaj J (2019). Somatic embryogenesis in selected conifer trees
* Pinus nigra
* Arn. and Abies hybrids. Front Plant Sci.

[60] Vannini GL, Poli F (1983). Binucleation and abnormal chromosome distribution in *Euglena gracilis* cells treated with dimethyl sulfoxide. Protoplasma.

[61] Aronen TS, Krajnakova J, Häggman HM, Ryynänen LA (1999). Genetic fidelity of cryopreserved embryogenic cultures of open-pollinated *Abies cephalonica*. Plant Sci.

[62] Etienne H, Bertrand B (2003). Somaclonal variation in *Coffea arabica*: Effects of genotype and embryogenic cell suspension age on frequency and phenotype of variants. Tree Physiol.

[63] Nawrot-Chorabik K, Sitko K (2014). The effect of abscic acid and dimethyl sulfoxide and different temperatures on the cryopreservation process of *Abies nordmanniana* embryogenic callus. Phyton-Ann REI Bot.

[64] Chen GG, Ren L, Zhang J, Reed BM, Zhang D, Shen XH (2015). Cryopreservation affects ROS-induced oxidative stress and antioxidant response in *Arabidopsis* seedlings. Cryobiology..

[65] Li BL, Zhang M, Wang XZ, Jiang XR, Liu Y (2019). The role of hydrogen peroxide in cryopreservation of cockscomb (*Celosia plumosa*) seedlings. Acta Hortic.

[66] Salaj T, Panis B, Swennen R, Salaj J (2007). Cryopreservation of embryogenic tissues of *Pinus nigra* Arn. by a slow freezing method. Cryo-Lett.

[67] Salaj T, Matušíková I, Swennen R, Panis B, Salaj J (2012). Long-term maintenance of Pinus nigra embryogenic cultures through cryopreservation. Acta Physiol Plant.

